# Use of Synchronous Digital Health Technologies for the Care of Children With Special Health Care Needs and Their Families: Scoping Review

**DOI:** 10.2196/15106

**Published:** 2019-11-21

**Authors:** Marissa Bird, Lin Li, Carley Ouellette, Kylie Hopkins, Michael H McGillion, Nancy Carter

**Affiliations:** 1 School of Nursing McMaster University Hamilton, ON Canada; 2 McMaster Children's Hospital Hamilton, ON Canada; 3 Population Health Research Institute Hamilton Health Sciences Hamilton, ON Canada

**Keywords:** pediatrics, scoping review, digital health, children with special health care needs, asthma, congenital heart disease, palliative care, co-design

## Abstract

**Background:**

Use of synchronous digital health technologies for care delivery to children with special health care needs (having a chronic physical, behavioral, developmental, or emotional condition in combination with high resource use) and their families at home has shown promise for improving outcomes and increasing access to care for this medically fragile and resource-intensive population. However, a comprehensive description of the various models of synchronous home digital health interventions does not exist, nor has the impact of such interventions been summarized to date.

**Objective:**

We aim to describe the various models of synchronous home digital health that have been used in pediatric populations with special health care needs, their outcomes, and implementation barriers.

**Methods:**

A systematic scoping review of the literature was conducted, guided by the Arksey and O’Malley Scoping Review Framework. MEDLINE, CINAHL, and EMBASE databases were searched from inception to June 2018, and the reference lists of the included systematic reviews and high-impact journals were hand-searched.

**Results:**

A total of 38 articles were included in this review. Interventional articles are described as feasibility studies, studies that aim to provide direct care to children with special health care needs, and studies that aim to support family members to deliver care to children with special health care needs. End-user involvement in the design and implementation of studies is evaluated using a human-centered design framework, and factors affecting the implementation of digital health programs are discussed in relation to technological, human, and systems factors.

**Conclusions:**

The use of digital health to care for children with special health care needs presents an opportunity to leverage the capacity of technology to connect patients and their families to much-needed care from expert health care providers while avoiding the expenses and potential harms of the hospital-based care system. Strategies to scale and spread pilot studies, such as involving end users in the co-design techniques, are needed to optimize digital health programs for children with special health care needs.

## Introduction

### Background

Advances in neonatal and pediatric care for complex medical conditions have contributed to the increased survival of children who live with chronic health care needs [1]. Although definitions of this group vary, children with special health care needs are generally considered to be those with or at risk for chronic physical, developmental, behavioral, or emotional conditions, often requiring substantial use of health and social services [2,3]. In the United States, the prevalence of children with special health care needs is estimated to be 19.8% of the pediatric population [4]. Canadian provincial administrative data report a similar prevalence rate of 17.5% [5].

Children with special health care needs often require care from specialists, typically located in urban tertiary centers [6]. In between specialist visits, children with special health care needs frequently experience the need for urgent care, often delivered by health care providers unfamiliar with their complex histories, intersecting conditions, and intricate care regimens [7]. This scenario often leads to extemporized courses of clinical management as well as recurrent emergency department visits and hospital admissions [8]. Such unpredictability confers vulnerability for children with special health care needs in terms of exposure to medical errors and other nosocomial harms such as infection [9].

Although children with special health care needs comprise less than 20% of the pediatric population in the United States, they account for 41% of total pediatric health expenditures [10]. Substantial time and resources are also contributed by families who care for children with special health care needs, estimated at 1.5 billion hours of care in the United States in 2015 [11]. Were these care hours provided by health care aides, the cost would approximate to US $35.7 billion or US $6400 per child [11]. Foregone income due to caregiving responsibilities in the home, as well as out-of-pocket expenses for parent and family members, add to the cost burden. Losses in parental earnings are estimated at US $3200 per child per year, and annual out-of-pocket expenses have been documented at over US $1000 per year in 20%-25% of children with special health care needs families [12].

### Prior Work

Recent attention has been given to synchronous digital health technologies, designed to increase access for patients and families to clinical teams in real time from their homes. Synchronous digital health technologies refer to the use of audio, video, and health information interfaces to facilitate the provision of health care remotely, in real time [13]. Both randomized and nonrandomized studies of digital health interventions in children with special health care needs to date have shown improved clinical, economic, and quality of life outcomes [14-16]. Synchronous digital health technologies have also been documented to improve parental caregiver outcomes such as quality of life, psychological health, satisfaction with care, and social support. One systematic review reported that 62 of 65 studies (95%) of synchronous digital health technologies observed significant improvements in these outcomes for caregivers of children and adults with chronic and degenerative diseases [17].

A national survey in the United States documented 51 digital health programs providing care to pediatric populations [18], supporting the momentum for such programs. At this time, the number of existing digital health pediatrics programs in Canada is unknown. Although the evidence base in support of the effectiveness of pediatric synchronous digital health interventions is growing [16,19-21], a comprehensive description of the ways in which synchronous home digital health solutions are used to care for children with special health care needs and support for their families is not yet documented.

### Purpose and Objectives

The purpose of this review is to summarize the current body of literature in order to describe how synchronous digital health technologies are used in the care of children with special health care needs and their families and to provide practical information for health care decision makers, considering digital health program implementation or expansion.

## Methods

### Scoping Review Phases

A scoping review was undertaken to allow for examination of the breadth of research activity on the design of digital health interventions for children with special health care needs, implementation, uptake, and evaluation of these programs as well as health care provider and family involvement in digital health solutions. Levac and colleagues’ [22] revision of Arksey and O’Malley’s [23] original methodology was used to conduct this work in five phases: (1) identifying the research question; (2) identifying relevant studies; (3) study selection; (4) charting the data; and (5) collating, summarizing, and reporting the results.

### Search Strategy

The search strategy was designed to capture a wide breadth of literature related to the research question, irrespective of study design. We included any type of article, book, dissertation, or report describing the use of synchronous digital health technologies to provide direct care to children with special health care needs or aimed at parents or caregivers with the intention of affecting outcomes in children. With the assistance of a librarian, a comprehensive search of the MEDLINE, CINAHL, and EMBASE databases was conducted by the first author (MB). Subject headings and keywords were used to locate articles describing the use of digital health in home settings for pediatric populations. The indexes of four key journals were also hand-searched for relevant articles. The initial literature search was run on June 30, 2018, with no date, age, or geographical limits set in order to increase the breadth of results. During the screening and data extraction phases, reference lists of highly relevant studies and reviews were scanned, and additional studies were screened for inclusion.

### Inclusion and Exclusion Criteria and the Review Process

Inclusion was based on four criteria: (1) the population of interest was children (<18 years) or children’s caregivers; (2) the population met the definition of children with special health care needs articulated by Newachek et al [24], ie, having a chronic physical, behavioral, developmental, or emotional condition in combination with high resource use; (3) care for the child was ongoing and occurring in the home setting; and (4) care for the child was delivered by synchronous digital health. All studies included at least one synchronous intervention element (eg, real-time phone call or video visit.). However, included studies could feature multifaceted interventions that included nonsynchronous components as well. Papers were excluded if they were not published in English, no full text was available, or if they were published prior to 2008 in order to ensure that the interventions described were relevant to stakeholders today. In accordance with scoping review methodology [22,23], no quality assessments were completed on the selected articles.

### Screening and Data Extraction

A two-stage screening process using screening forms developed by the team was employed for this review. Prior to screening, a validation test of the title and abstract screening tool was first completed by two authors (MB and NC). Validation screening resulted in 90% agreement, with conflicts resolved through discussion and consensus between authors. After refinement of the screening tool, title and abstract screening was completed by one author (MB). Prior to full-text screening, all authors met to arrive at a consensus on the inclusion criteria. Test screening of three articles per author was performed, and discrepancies were resolved via email communication. Each author was then assigned articles to screen and extract data from using a standardized survey template. Authors were in frequent communication during the screening process, and weekly emails with updates, group questions, and discrepancies were circulated to ensure consistency.

### Analysis

#### Frameworks Used

Our interest in providing decision makers with relevant information related to digital health program implementation or expansion prompted us to extract and analyze practical considerations of these applications. To this end, we analyzed digital health intervention characteristics, end-user involvement (patients, families, and health care providers) in digital health intervention design, and barriers to implementation. Data extracted from relevant articles were downloaded into Excel (Microsoft Corporation, Redmond, Washington) files and reviewed by research team members. We used two frameworks to guide analysis: Data from feasibility studies are presented using a framework by Bowen and colleagues [25], and end-user involvement in co-design and implementation was evaluated using the Human-Centered Design framework from IDEO [26]. The two frameworks are described briefly below.

#### Feasibility

Our use of the term “feasibility” is broad in nature, in keeping with work by Bowen and colleagues [25], suggesting that feasibility trials encompass any study that assists investigators to prepare for a full-scale trial of intervention effectiveness. Using this definition, feasibility outcomes may be grouped into eight general areas of focus, which include acceptability (intervention recipient feedback), demand (intervention use), implementation (success of intervention deployment), practicality (interference with resource use), adaptation (necessary modifications), integration (fit of intervention to context), expansion (intervention applications to new context), and limited-efficacy testing (preliminary outcomes) [25].

#### Human-Centered Design

We sought out information from all papers related to the inclusion of end users in digital health intervention design and implementation using the IDEO Framework as a guide to this data extraction. Consisting of a six-stage, iterative cycle, the IDEO Framework aims to increase the relevance and appropriateness of interventions [26]. End users are included in the stages of observation (understanding the end user), ideation (brainstorming ideas), prototyping (creating rough intervention mock-ups), user feedback (soliciting input from end-users), iteration (intervention refinement), and implementation (deployment into practice) [26]. In the health care sector, the IDEO Human-Centered Design framework has been used to generate solutions such as helping patients remember to take their prescription medications and communicating messages of support to women recovering from surgical procedures [27]. Finally, consideration was given to issues of digital health implementation in relation to technological, human, and system-level factors.

## Results

### Numbers, Sources, and Types of Papers

**Figure 1:**
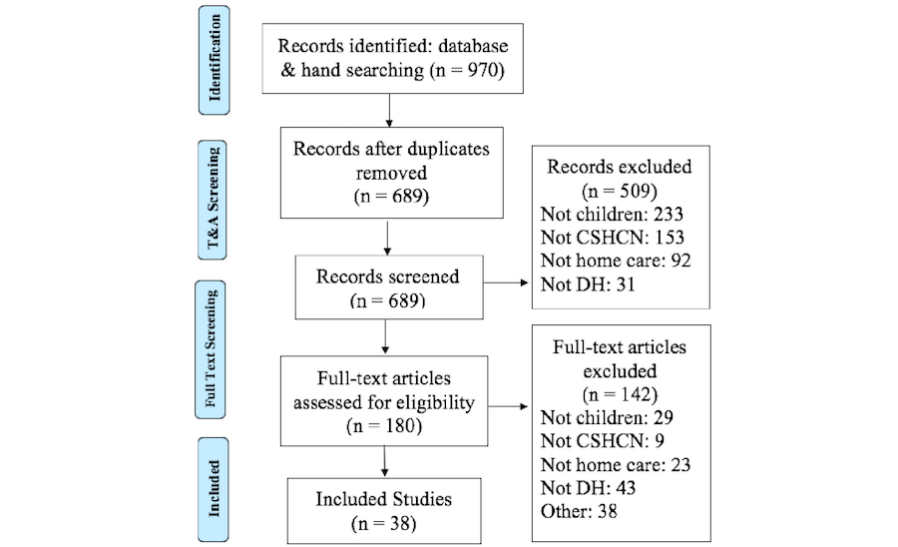
Preferred Reporting Items for Systematic Reviews and Meta-Analyses flow diagram. CSHCN: children with special health care needs; DH: digital health; T&A: title and abstract.

Results of the screening process and overall yield of papers are presented in Figure 1. Of the 38 papers included in the review, as shown in Table 1, 50% originated in the United States—an expected result, given the size and population base. Eleven articles originated in Australia, where the use of digital health may represent a solution to timely care delivery for the country’s large rural and remote population.

Table 2 depicts the variation in study design, as reported by the authors. The majority of the papers reported on evaluation of digital health initiatives through feasibility studies (n=12), program evaluations (n=8), randomized controlled (n=6), nonrandomized controlled trial (n=3), mixed methods (n=1), and cohort studies (n=1).

**Table 1:** Yield of papers by country of origin.

Country of origin	Number of papers	References
United States	19	[15-17,19,28-42]
Australia	11	[7,14,43-51]
United Kingdom	3	[52-54]
Germany	1	[55]
Israel	1	[56]
The Netherlands	1	[57]
New Zealand	1	[58]
Scotland	1	[59]

**Table 2:** Yield of papers by stated research method (N=38).

Research method	Number of papers	References
Feasibility studies (n=12)	12	[30,31,43,45-47,49,51,53,56-58]
Program evaluation (n=8)	8	[7,15,29,33,38,40,48,50]
Randomized controlled trial (n=6)	6	[16,19,32,35,54,55]
Nonrandomized controlled trial (n=3)	3	[28,37,44]
Discussion paper (n=2)	2	[34,59]
Review (n=2)	2	[17,42]
Cost minimization analysis (n=1)	1	[14]
Descriptive (n=1)	1	[36]
Mixed methods (n=1)	1	[52]
Cohort (n=1)	1	[41]
Qualitative (n=1)	1	[39]

### Studies Reporting on Digital Health Interventions

A major focus of this review was on empirical studies that evaluated the use of digital health in caring for children and families. A large number of the empirical studies included were feasibility trials, leading us to report these separately from full-scale studies. Here, we first describe feasibility trials and then studies that used digital health interventions to provide direct care to children with special health care needs (such as employing video consultations for physical assessments), followed by interventions aimed at supporting families to care for children at home. Where possible, we have included information on published statistical results; however, many studies were performed with small samples, and therefore, the results were not analyzed for statistical significance.

### Feasibility Studies

Table 3 provides details of the feasibility studies using digital health interventions. Based on Bowen and colleagues’ [25] definition of feasibility studies, we identified 12 articles that reported feasibility-related outcomes. Of note, five of these studies were conducted with hematology/oncology/palliative care populations, whereas the remaining interventions targeted diverse disease groups. One intervention used telephone calls and a blog for communication [58], another used “Skype” and “WhatsApp” for video chats and text messaging [56], and all other studies utilized video formats with either embedded audio or separate telephone audio. There was a wide range of uses for digital health, including assessing acute clinical issues, providing routine care and follow-up, facilitating case conferences, providing psychosocial support, delivering therapy, and monitoring progress and adherence.

Among the included studies, six of the eight dimensions of feasibility were measured, and these outcomes are reported in Table 3. Ten studies looked at *acceptability*, with seven studies measuring family-reported acceptability [31,45-47,53,56,57], and five studies measuring health care provider acceptability [45-47,51,53]. Overall, most families and health care providers reported high satisfaction with digital health interventions and found the equipment to be easy to use. The *demand* for digital health was reported in seven studies by describing the number and length of calls made over the study period [31,43,45,46,49,53,57]. Two of these articles also measured changes in demand over time, with both studies observing an increase in the utilization of digital health over the study period [49,57]. A total of seven studies reported *implementation* and *integration* issues in the form of technical difficulties [30,45-47,51,53,56]. These technical problems were both human related (eg, confusion with using equipment) and technology related (eg, firewall settings, poor internet coverage in remote areas, and bandwidth limitations). In terms of *practicality*, three studies conducted cost analyses [45,49], and two studies found that patient and staff availability, workloads, and scheduling influenced how the intervention was implemented [45,56].

Four studies conducted *limited-efficacy testing* of their interventions [31,53,56,58]. Gur and colleagues [56] piloted the use of text messaging and video chats with individuals with cystic fibrosis, but found no statistically significant differences in measured outcomes between the control and intervention groups. The remaining three studies did not have control groups but reported benefits of improved child functional outcomes [58], reduced parental anxiety (median State and Trait Anxiety Inventory score reduction: 6 points; *P*<.05) [53], and prevention of health care visits/admissions [31]. Among all the feasibility studies identified, none adapted a previously established program or reported on outcomes related to the expansion of an already successful intervention. Additionally, four studies led to future publications describing larger-scale interventions [30,31,43,53].

**Table 3:** Feasibility studies.

Study identifiers: first author (year), country (sample size) [reference}	Study purpose: objectives, uses	Intervention characteristics: technology used, diagnosis of sample, health care providers	Feasibility outcomes: acceptability, adaptation, demand, integration, implementation, expansion, practicality, limited-efficacy testing
Ludikhuize (2016), Netherlands (n=21) [57]	Determine feasibility of adding video to phone consultations in order to reduce the need for patients to travel long distances Assessment and follow-up of acute bleeding	Home computer with webcam or tablet/phone to hemophilia treatment center Hemophilia Registered nurse, physician - specialist	Acceptability: high satisfaction with video quality. Patients/parents reported adding video led to better consultations; health care providers reported video helped them assess severity of bleeding. Demand: 29 phone or video consultations took place over 13 months with 10 of 21 enrolled patients. Use of video consultations increased over the trial period.
Katalinic (2013), Australia (n=14) [51]	Improve access to services, self-management of health conditions and health education; reduce social isolation for rural and remote patients. Clinical review, case conferences, education and bereavement follow-up	Home tablet (iPad) to clinical service 4 clinical services, including pediatric palliative care APN^a^, physician (specialist), occupational therapist, SW^b^	Acceptability: high usability ratings; portable and customizable Implementation: low-cost and little set-up required. Complex licensing and application purchasing; difficulties with customizing implementation. Technical problems: firewall outages, poor internet coverage, integration issues, bandwidth limitations
Bradford (2010), Australia (n=2) [43]	Describe two case studies illustrating the value of home telemedicine Clinical management, anticipatory guidance, and psychosocial support	Computer and webcam (video only) and phone (audio) to telehealth center Palliative care Registered nurse, physician (specialist), “hospital clown doctors”	Demand: case 1 had 37 calls lasting 10-20 minutes over 7 months (23 with Clown doctors and 15 with specialist team). Case 2 had one 45-minute call.
Bensink (2009), Australia (n=11) [46]	Determine acceptability of videotelephony for families receiving pediatric palliative care. Add video to existing telephone support provided by specialist nurses in the hospital to regional and remote families.	Home computer with webcam (video) and telephone (audio), linked to a computer, webcam, audio-conferencing system in the hospital. Palliative care Specialist registered nurse, physician (specialist), SW	Acceptability: 92% participant consent rate; high nurse satisfaction with video and audio quality. Demand: 25 calls with 7 of the 11 consenting families. Implementation: Technical problems were human related (*n*=3) and technology related (n=1). Practicality: cost analysis reported.
Bensink (2008), Australia (n=8) [45]	Test the feasibility of providing videotelephone-based discharge support to families with a child newly diagnosed with cancer. Provide practical, emotional, and symptom support to families.	Home computer with webcam (video) and home or mobile phone (audio) Oncology APN, SW	Acceptability: high family satisfaction with service; high nurse satisfaction with audio and video quality. Demand: 20 calls were made with 7 families over a 3-month period, totaling 400 minutes. Implementation: problems with video were human related (n=1) and technical (n=2). Practicality: calls required organization around ward workflows.
Gur (2017), Israel (n=18) [56]	Assess the feasibility of using WhatsApp and Skype to improve treatment adherence by enhancing communications between patients/families and health care providers. Evaluation and encouragement of treatment adherence, addressing barriers to adherence.	Text messaging (WhatsApp) and video (Skype) CF^c^ Registered nurse, physician, physiotherapist, dietician, psychologist, SW	Acceptability: patients were very satisfied with the intervention. Practicality: scheduling difficulties. Integration: technical issues with wireless internet in some remote areas. Limited-efficacy testing: No difference in CF-related self-rated health, CF-specific knowledge, treatment adherence, or patient-rated relations with their teams between groups.
Casavant (2014), US (n=14) [31]	Investigate whether telemedicine is feasible, affects confidence of families in clinical management, and supports clinical decision-making. Routine health care visits, follow-up of clinical problems, and urgent assessment when home visit not possible.	Family’s existing computer with webcam to study team Children with chronic respiratory insufficiency on home ventilation Physician (specialist), respiratory therapist, APN, SW, program administrator	Acceptability: families reported intervention ease of use, high audio and video quality, and no added costs. Families perceived health care providers were better able to assess their child and received better overall clinical management compared to phone Demand: 27 video conferences with 14 families over 9 months; 15 calls were for routine care, 10 for follow-up of specific issues, and 2 for acute illness. Limited-efficacy testing: prevented 23 clinic visits; 3 emergency department visits, and 1 hospital admission.
Jury (2014), Australia (n=not reported) [49]	Increase convenience for families, reduce physician travel, provide additional services, conserve physical space, and provide more equitable health care access. Follow-up, outreach for remote communities.	Web-based video-consultations 37 departments at The Royal Children’s Hospital in Melbourne have provided video-consultations Mixed health care provider groups	Demand: increase in consultations (from 14/month to 49/month); 92% of departments had provided at least one video consultation. Practicality: 65 billed appointments per month are needed to fund a coordinator. 36% of booked appointments were not billed to Medicare.
Constantinescu (2012), Australia (n=17) [47]	Provide access to therapy and reduced costs for children and families living in rural and remote areas. Weekly planning and audio-verbal therapy sessions.	Computer-based videoconferencing (Skype) Children with hearing loss Auditory-verbal therapist	Acceptability: High parental and therapist satisfaction; parents and therapists reported moderate audio and video quality; parents reported more technical difficulties and less comfort with technology than therapists.
Miyahara (2009), New Zealand (n=7) [58]	Develop and implement a family-focused intervention program to improve the coordination of children with developmental coordination disorder. Progress monitoring of developmental coordination disorder.	Workbook, DVDs, weekly telephone consultations, and a blog Children with developmental coordination disorder Physiotherapist	Acceptability: parents voiced appreciation for the weekly telephone consultations and reported that telephone consultations encouraged program adherence. Limited-efficacy testing: all families reported improvements in their children’s functional motor skills.
Cady (2008), US (n=5) [30]	Evaluate feasibility of videoconferencing between study office and family homes. Assessment, management of acute and chronic conditions, dissemination of health information, coordination of services.	Webcam (supplied) with family’s own computer to study nurse Children with medical complexities APN	Acceptability: unscheduled video visits were rated by nurses as providing more information than a telephone call. Implementation: initial connections failed due to firewall settings—case-by-case resolution needed. Integration: video quality in rural settings was insufficient for clinical assessment.
McCrossan (2008), UK (n=5) [53]	Investigate the feasibility of videoconferencing using broadband transmission. Assessment and provision of home support and advice after hospital discharge.	Twice weekly videoconferences with pulse oximeter for 10 weeks Complex congenital heart disease Clinician (not specified)	Acceptability: “good” to “very good” ratings by health care providers and parents. Demand: 78 video conferences over a 6-month period with 5 patients. Implementation: technical problems related to connectivity and video quality occurred in 10 videoconferences (13%). Limited-efficacy testing: reduction in parental anxiety following video consultations.

^a^APN: advanced practice nurse.

^b^SW: social worker.

^c^CF: cystic fibrosis.

### Interventions to Provide Direct Care via Digital Health

Ten articles representing seven studies described the use of digital health with children with special health care needs for the purposes of providing direct patient care or replacing in-person assessments (Table 4). Of these, six articles (four studies) examined digital health interventions for children with medical complexities [7,15,16,19,29,33], two articles (one study) focused on palliative care [14,44], one article focused on asthma [38], and one article focused on children with congenital heart disease [33]. Telephone was an interventional component in all studies; the next most commonly employed technologies were video [16,19,54] and email [33]. The makeup of digital health teams varied between studies: Some interventions were delivered by a single group of practitioners such as registered nurses [7,38] or advanced practice nurses [15,16,19,29], while others involved a multidisciplinary team [14,33,44]. One study did not specify the profession of the consultant involved in the intervention [54].

Studies that examined children with special health care needs–related outcomes had mixed results, while studies that examined family-related outcomes reported mainly positive results. Positive outcomes for children with special health care needs were constituted by parent-reported decreases in hospitalizations and quicker recovery from illness [29], reductions in unplanned hospitalizations (year 1 mean number of unplanned hospitalizations per child: 1.7; year 2 mean number of unplanned hospitalizations per child: 0.8; *P*<.007) [15], reduced health care resource use (37% lower in the video conferencing group compared to the control groups; *P*<.05) [54], and improved asthma severity scores [38]. In contrast, two studies found no change in emergency department visits (18.4% enrolled patients presented to the emergency department per month in 2003 and 15.0% per month in 2006; *P*=.41) or hospital admissions (8.0% of enrolled patients hospitalized per month in 2003 and 7.3% hospitalized per month in 2006; *P*=.67) [7], and no significant differences in health-related quality of life as measured by the PedsQL based on analysis of variance scores (*F*=0.90; *P*=.41) [16] for children with special health care needs. Family members reported overall high satisfaction scores with digital health interventions, for example, average scores reported were 8.3/10 [7], and 9.3/10 [33]. Parents participating in the intervention arm of a digital health study rated their satisfaction with their child’s personal doctor (*P*=.001) and level of care coordination (*P*=.03) as significantly better than control groups based on the Consumer Assessment of Healthcare Providers and Systems Clinician and Group survey [19], and in an additional study, parents perceived availability of digital health to be “very important” in assisting them in managing their child’s condition at home [29]. However, using descriptive analysis, Bradford and colleagues [44] found no change in caregiver quality of life in parents of children receiving palliative care via digital health.

**Table 4:** Interventions to provide direct patient care via digital health.

Study identifiers: first author (year), country (sample size) [reference}	Study purpose: objectives	Intervention components: technology used, patient population, intervention, health care providers	Reported or perceived outcomes
Graham (2017), US (n=320) [33]	Describe the utilization and satisfaction of a program with 24/7 family-driven access to health care teams with the aim of providing comprehensive, individually tailored care to children with CRI^a^	Telephone and email Children with CRI Home and clinic visits, care coordination, and ongoing access to physicians Physician (specialist), respiratory therapist, APN^b^, SW^c^, program administrator	SO^d^: Telephone calls accounted for 40%-50% of patient encounters over a 3-year study period, but telemedicine only accounted for 0.3%-1.1% of all visits. Average numbers of encounters per patient per year increased over the study period (increase mainly attributable to telephone and email communication); decrease in in-person visits over study period. FO^e^: Family satisfaction rating of intervention was 9.3/10.
Cady (2014), US (n=27) [29] and Cady (2009), US (n=43) [15]	Describe the attributes and effects of an APN-administered care coordination program for children with medical complexities and their families	Telephone Children with moderate/high intensity health care needs Case management and care coordination Primary care provider, APN, RN^f^ coordinator, physician (specialist), support staff	PO^g^: ≥80% of parents perceived their child to be hospitalized less frequently and recover from illness faster compared to before the program [29]. SO: Over 3 years, the number of care coordination episodes tripled, with significant increase between years 1 and 2 (*P*<.001) [29]; 48% of episodes were initiated for acute and chronic condition management [29]; statistically significant reduction in unplanned hospitalizations between years 1 and 2 (*P*<.007), with stable rates of planned hospitalizations (*P*=.14) [15] FO: 80% of parents were more comfortable being discharged home from the hospital [29].
Looman (2015), US, (n=148) [19] and Looman (2018), US (n=163) [16]	Examine the effects of adding a high-intensity, APN-delivered digital health care coordination intervention within an existing medical home model	Telephone or video Children with medical complexities and their families High-intensity care coordination APNs	FO: Telephone group had significantly higher satisfaction scores on the global health care rating category (*P*<.05) and the health care provider communication measure (*P*<.01) compared to the control group [19]; parents rated care coordination and children’s personal doctors as significantly better in both the video and telephone intervention groups, compared to the control group (*P*<.05) [19]. Intervention did not significantly improve child health-related quality of life or disease burden on family (all *P*>.05) [16].
Sutton (2008), Australia (n=220) [7]	Determine if continuous mobile phone access to ED^h^ RNs can increase families' capacities to manage care of child at home and decrease ED visits and ED length of stay	Telephone Children with medical complexities Enrollment in a program with access to advice and rapid emergency department care ED RNs with extensive triage and resuscitation experience	FO: Family satisfaction with the program was 8.3/10. SO: Phone calls increased from an average of 0.24 calls/participant in 2003 to 0.3 calls/participant in 2006, 60% of which were after hours; no significant difference in the number of ED presentations as a percentage of enrolled patients (*P*=.41), number of hospital admissions as a percentage of enrolled patients (*P*=.67), or hospital admission rates after ED presentation (*P*=.70). Approximate cost of the program/child was AU $750 (£292; USD $511)/year.
Bradford (2014), Australia (n=not reported) [14] and Bradford (2012), Australia (n=14) [44]	Measure the effects of a home digital health program for pediatric palliative care consultations on caregiver quality of life. Compare in-person with video palliative care consultations	Telephone and video Children in palliative care Specialist pediatric palliative care home video consultations to advise on symptom management, care planning, and emotional support. RN consultant, physician (specialist), project officer	FO: Descriptive analysis showed no differences in caregiver quality-of-life scores between intervention and control groups [44]. SO: digital health intervention saves AU $244 (USD $166)/year to AU $7598 (USD $5182)/year compared to outpatient or home visit appointments requiring road-only travel. Digital health intervention saves AU $23,758 (USD $16,205)/year to AU $45,925 (USD $31,330)/year compared to outpatient or home visit appointments requiring air travel [14].
Nelson (2009), US, (n=not reported) [38]	Describe a severity-based nurse-administered asthma management protocol administered to children/families at home via telephone	Telephone Children with asthma Access to a nurse-staffed call center after hours, weekends, and holidays for care advice and treatment recommendation RNs	PO: Urgent calls had improved severity scores at follow-up; 28% of patients recommended home treatment were referred to ED at follow-up. FO: 95% parents reported implementing recommended home treatments.
McCrossan (2012), UK (n=83) [54]	Evaluate a digital health intervention for clinical utility and intervention quality, and determine impacts on health care resource use	Telephone or video Children with congenital heart disease Video or telephone consultations 1-2 times per week were conducted to assess patients with congenital heart disease and address parents’ questions. Clinician (not specified)	PO: Probability of being admitted to hospital was significantly less in the video group compared with the telephone and control groups (*P*=.004). FO: Parents reported video consultations were superior to telephone consultations with regard to facilitating communication and overall benefit (*P*=.001). SO: Video consultation group used 37% fewer health care resources than either telephone or usual care groups (*P*<.001). HPO^i^: Health care providers significantly more likely to report they could address parents’ concerns in video versus telephone groups (*P*=.01).

^a^CRI:. chronic respiratory insufficiency

^b^APN: advanced practice nurse.

^c^SW: social worker.

^d^SO: system outcomes.

^e^FO: family outcomes.

^f^RN: registered nurse.

^g^PO: patient outcomes.

^h^ED: emergency department.

^i^HPO: health care provider outcomes.

### Interventions to Teach and Support Parents and Families

Seven papers described digital health interventions intended to train or provide support to parents of children with special health care needs (Table 5). Four of these papers involved parents of children with autism spectrum disorder [28,37,40,41], two papers were focused on asthma [32,35], and one was focused on a mental health issue [55]. In four studies, behavior consultants or therapists used video to train parents of children with autism spectrum disorders to use autism specific interventions including applied behavioral analysis [28,37,40,41]. Reported outcomes of these interventions include reduction in problem behavior [37,40] and gains in communication skills for children [28]. For example, Lindgren and colleagues [37] found a mean reduction in problem behavior of over 90% for children with autism treated by specialists in their homes (mean reduction: 95.76%), by telehealth in a clinic setting (mean reduction: 91.00%), and via telehealth in their homes (mean reduction: 97.27%). Between-group differences based on analysis of variance scores were significant (*P*=.07).

Two papers used telephone consultation to support and train parents of children with asthma [32,35], with mixed outcomes reported. Neither study reported any benefit in patient outcomes: Gustafson and colleagues [35] found no difference in medication adherence (*P*=.76) or number of symptom-free days for children (*P*>.99), while Garbutt and colleagues [32] found no improvements in either children’s quality of life as measured by the Pediatric Asthma Quality of Life Questionnaire (between group difference: –0.17; 95% CI −0.47 to 0.12) or number of urgent events per year (between group difference: 1.15; 95% CI 0.82-1.61). However, at the family level, they reported that parental quality of life (measured using the Pediatric Asthma Caregiver’s Quality of Life Questionnaire) improved with an asthma coaching program (between-group difference 0.38; CI 0.14-0.63).

Kierfeld and colleagues [55] used a telephone intervention with minimal therapist contact to train parents of children with externalizing problem behaviors. Results included improvements in the treatment group in problem behaviors, as measured by analysis of variance (F_1,44_=21.14, *P*<.001, d_diff_=1.22), parenting strategies (F_1,43_=9.43, *P*=.002, d_diff_=0.92), and parenting-related strains (F_1,43_=12.28, *P*<.001, d_diff_=1.03) [55].

**Table 5:** Interventions to train or support parents to deliver care (n=7).

Study identifiers: first author (year), country (sample size) [reference}	Study purpose: objectives	Intervention components: technology used, patient population, intervention, health care providers	Reported or perceived outcomes
Lindgren (2016), US (n=107) [37]	Determine whether challenging behavior in children with autism can be treated successfully at lower cost by using telehealth to train parents to implement applied behavior analysis	Video (Skype) through the telehealth center Parents of children with autism spectrum disorder Weekly 60 minutes sessions where parents were coached to perform functional analysis and functional communication training Behavior analysts or advanced graduate students	PO^a^: reduction in problem behavior achieved but no different than traditional method (*P*=.74). SO^b^: reduction of costs related to treatment compared to in-home therapy (for staff salaries and travel, facilities, and family costs including telehealth equipment, mileage, and time) (*P*<.01).
Suess (2014), US, (n=parents of 3 children) [40]	Evaluate the fidelity with which parents of children with autism spectrum disorders implemented treatment procedures and the types of fidelity errors they made during coached and independent trials	Video and Skype connection with telehealth center Parents of children with autism spectrum disorder Two sessions of didactic training, parent manual, weekly remote consultation, while parents implemented Functional Communication Training procedures Behavioral consultant (psychology doctoral student experienced in behavior assessments and treatments)	PO: all children showed substantial reductions in problem behavior during the final treatment trials and especially during the coached trials. FO^c^: no consistent differences present in measurements of intervention implementation fidelity by parents across coached and independent trials.
Vismara (2013), US (n=8 families) [41]	Teach parents to implement autism-specific interventions	Video and self-guided website Parents of children with autism spectrum disorder Weekly 1.5-hour parent coaching sessions for 12 weeks with 3-month follow-up Therapist with extensive training	PO: overall improvement in rates of functional verbal utterances and nonverbal joint attention initiations, increased production and comprehension of words and gestures. FO: steady gains in parental intervention skills, engagement style, and fidelity of intervention implementation.
Baharav (2010), US (n=2) [28]	Assess the use of technology and telepractice as a tool for coaching parents of children with autism spectrum disorders.	Home laptop with Web camera and health care provider laptop Parents of children with autism spectrum disorder Weekly 50-minute home-based and 50-minute clinic sessions over 6 weeks Speech and language therapists	PO: Gains in some communication and interaction skills. FO: Parents report comfort with technology, willingness to continue to practicing strategies to deliver care to their child at home, and agree home services as valuable as those delivered by healthcare providers and would recommend to other patients
Gustafson (2012), US (n=301 parent-child dyads) [35]	Support and train parents and improve asthma control and medication adherence.	Telephone Parents of children with asthma Electronic health intervention with interactive tools and tailored content and monthly support from nurse case manager	PO: No significant difference in symptom-free days (**P*>.99*), or medication adherence (*P*=.76) between groups.
Garbutt (2010), US (n=362) [32]	Coach parents and children with asthma to improve disease-related quality of life and reduce incidence of asthma episodes requiring urgent care.	Telephone from call center Parents of children with asthma 12-month coaching program to provide education and support Call center RNs^d^ with pediatric and asthma telephone care experience	PO: No change in children’s quality of life (95% CI −0.47 to 0.12) or number of urgent events per year (1.15; 95% CI 0.82 to 1.61). FO: Significant improvement in parental quality of life with coaching program compared to control group (difference: 0.38; 95% CI 0.14-0.63). SO: no change in number of urgent events per year (difference: 1.15; 95% CI 0.82-1.61)
Kierfeld (2013), Germany (n=48 families) [55]	Support and train parents of children with externalizing problem behavior to administer interventions with minimal therapist contact	Telephone Parents of children with externalizing problem behavior Self-help book and weekly phone calls (average 20 min) to enhance motivation by reviewing key concepts covered in the self-help book Child psychologist trained and supervised by senior child psychologist	PO: Improvements in parent-reported externalizing behaviors (F_1,44_=21.14, *P*<.001, d_diff_=1.22), and internalizing child problem behavior (F_1,44_=13.52, *P*<.001, d_diff_=1.01) FO: Improvements in problem parenting strategies (F_1,43_=9.43, *P*=.002, d_diff_=0.92, and parenting-related strains (F_1,43_=12.28, *P*<.001, d_diff_=1.03).

^a^PO: patient outcomes.

^b^SO: system outcomes.

^c^FO: family outcomes.

^d^RN: registered nurse.

### Family and Health Care Provider Involvement in Design of Digital Health Interventions

Across the body of included literature, there were few studies that explicitly included families and health care providers (intervention end-users) in the design and implementation of digital health interventions. However, a few key examples showcased end-user involvement, most commonly, in the early stages of intervention design such as the *observation* or *ideation* phases, as well as by garnering *user feedback*.

In one study by Miyahara and colleagues [58], the researchers actively involved families in the development, testing, and refinement of the intervention (*feedback* and *iteration*). An iterative process of two-way communication between the researchers and participants was used to evaluate and refine the intervention (a set of digital versatile discs, a workbook, and a website) throughout the study [58]. Authors reported that the impacts of end-user involvement increased participation in interventional components as well as the development of educational materials that were acceptable and useful to parents. Cady and colleagues [30] conducted a survey prior to initiating a videoconferencing intervention to find out what types of technologies were available to families (*observation*). Results of the survey supported that most families already had adequate home technology to support videoconferencing; however, apparent survey response bias led the researches to caution of a potential “digital divide” in access to technology between Caucasian and minority populations [30]. Finally, Sutton’s group [7] engaged in a formal parent survey and the collection of anecdotal feedback from parents, health care providers, and subspecialty staff about the current care model, which spurred the development of the intervention (*observation*). Researchers then developed a study advisory group, consisting of key stakeholders such as parents and a variety of health care providers (*ideation*). Although the exact responsibilities of the advisory group are unclear, the inclusion of an end-user advisory group can lend valuable insights into intervention content and structure, making interventions more user-friendly and feasible to implement [60].

### Factors Affecting Implementation of Digital Health Technologies

In addition to implementation challenges reported in the feasibility studies section, we also examined included studies for factors that may impact implementation. These factors, which we categorized as *technological, human,* or *system*, stem from family and health care provider perceptions as well as lessons learned by the researchers.

#### Technological Factors

Many studies reported encountering technical issues, which affected the implementation and acceptance of digital health interventions if the quality of videoconferencing or health care provider workflow patterns are disrupted [30,51]. For example, a barrier to videoconferencing was the limited availability of devices and broadband internet [57]. To overcome barriers to access, some interventions supplied equipment or internet services to families in varying capacities such as webcams, software packages, and computers on loan from the study with prepaid wireless connections [16,19,43,46,51,53,57]. These practical considerations are vital to acknowledge and plan for prior to digital health intervention deployment.

#### Human Factors

In general, patients, families, and providers were satisfied with digital health interventions and were open to learning how to use new technologies if they thought it would save them time [51]. However, digital health was not always appropriate, depending on the clinical use case. For example, Constaninescu [47] reported that therapists had difficulty engaging with younger children with hearing loss during videoconferencing appointments. Additional human factor barriers noted by Edirippulige and colleagues [48] were that social workers preferred in-person appointments to facilitate a personal connection with patients, and Seuss’ team [40] hypothesized that some parents may require face-to-face demonstrations of clinical skills for optimal treatment fidelity. With regard to human-technology interfaces, Casavant and colleagues [31] reported that the availability of real-time visual images was an important factor in decision making for health care providers treating children on home ventilator support, and a lack of visuals was cited as a concern for health care providers in two phone-only interventions [36,52]. Additionally, some studies cited barriers of scheduling, time constraints, and workload for both patients/families and health care providers [16,45,48,56,58]. Family commitment (ie, history of good attendance in clinic) and health care provider engagement were crucial for successful implementation of the digital health interventions, with health care provider engagement being facilitated by strong leadership and rapid resolution of problems [50,51].

#### System Factors

Several studies reported system factor barriers to digital health related to funding, such as difficulties in obtaining consent to bill and restrictions on who could be reimbursed for delivering digital health interventions [32,49]. Additionally, connectivity issues [30,45,51,53,56] and device interoperability between systems [30,51] were additional barriers. System factors that facilitated implementation include detailed planning, high-level support, standardization and education, and adequate administrative support [50,51].

## Discussion

### Principal Findings

In this scoping review, we sought to synthesize the current available evidence on the use of digital health to care for children with special health care needs and their families. Our results draw attention to gaps evident in the knowledge base in this area, including the few full-scale randomized trials testing such interventions, and the dearth of literature discussing the involvement of end-users in intervention design and implementation. Despite national studies such as the SPROUT survey in the United States reporting 22 dedicated pediatric digital health programs, and an additional 29 programs providing digital health to mixed adult and pediatric populations [18], published research on such programs remains scarce.

### Practical Considerations for Implementing Digital Health Technologies

This scoping review of the literature has demonstrated that digital health technologies have the potential to provide high-quality, effective interventions for children with special health care needs and their families in the convenience of their homes. Recent advances and widespread use of technology (eg, smartphones and tablets) have created an international landscape ready for implementation of digital health interventions. However, despite the pervasiveness of user-friendly technology, barriers to implementation continue to exist. Health care providers and health care administrators should consider the following implications when thinking about how to successfully implement digital health interventions.

Many of the included studies report the use of a digital health center or related infrastructure support, which may come with benefits such as having digital health–trained health care providers, dedicated technical support, and digital health–focused resources. Jury et al [50] reported the use of a website that contains staff and patient resources with how-to guides and troubleshooting material. However, other studies have demonstrated the effectiveness of interventions delivered by independent health care providers. For example, studies by Vismara and colleagues [41] and Baharav and Reiser [28] have shown therapeutic outcomes associated with interventions delivered by health care providers from their office computers. Although many studies reported technical issues such as connectivity or interoperability conflicts, it was often unclear whether dedicated ongoing technical support was available. When considering implementing digital health solutions, it is important to be aware of the type of infrastructure available, how technical support will be provided, and what effect program implementation will have on health care provider workflows. Explicit reporting of these vital factors in published journal articles or reports may assist in moving the field of digital health forward and achieving optimal digital health intervention integration into health systems.

In addition, some health care providers and administrators may be able to capitalize on available funding for the implementation of digital health interventions [50], which can assist in rapidly implementing or scaling a digital health program. To increase the uptake of digital health, decision makers should consider that funding must be available not only to set up infrastructure, but also to inform health care providers and families of digital health intervention availability on an ongoing basis, and to assist in day-to-day operational management of the program. For example, Jury et al [50] reported using a program manager and telehealth “champions” to facilitate implementation, promoting digital health to families, and referring general practitioners. The demand created by these promotional strategies may well neutralize the added costs of personnel involved in the digital health program in for-profit situations.

Finally, care equity deserves special consideration when implementing digital health interventions. For example, in rural and remote areas, poor internet connectivity may prove to be a significant challenge for digital health programs to overcome [51,56]. One method that was used when bandwidth was insufficient for high-quality video was to utilize the Internet for video, while using the phone line for audio [43,45,46]. Using this strategy, fluctuations in picture quality were mitigated by clear and reliable audio components, and the call was not entirely interrupted. An additional care equity point to consider when implementing digital health programs is families’ access to devices that are required for using digital health. Although some studies in this review excluded participants who did not have access to the required devices or sufficient internet speeds, others provided hardware or financial support to install high-speed internet. By excluding those who do not have access to devices or adequate internet, health care systems may be further marginalizing underresourced populations and exacerbate the “digital divide.” Crucial to the successful implementation of digital health interventions is finding solutions to mitigate barriers to access. Modern technology options such as tablets are cost-efficient and easy to use, albeit reliant on Web-based software. Conversely, videoconferencing units that utilize phone lines are more expensive and require more technical support but may be more suitable for remote regions. Regardless of the types of devices and connection used, having requisite supports in place to rapidly overcome technical and user-related barriers in the provision of digital health is essential for intervention uptake.

### Teaching Parents

A promising area of results of this review is the use of digital health to teach and support parents to deliver care to children with special health care needs. Across a multitude of clinical specialties, chronic disease self-management is heralded as promoting improved patient engagement and collaborative care [61]. For children with chronic conditions, self-management necessitates the involvement of parents or other caregivers to deliver requisite proactive planning, disease surveillance, and health maintenance. Lozano and Houtrow [62] highlight the need for children and youth with chronic conditions to participate in shared care management where possible while also allowing appropriate amounts of autonomy. The positive impacts of parental training noted in this review, particularly in studies examining the parental delivery of autism therapies, could have important implications for improving clinical outcomes and conserving health care resources.

### Co-Design of Digital Health Interventions

Literature in the field of intervention co-design reports that the concerns of health care practitioners and patients are often fundamentally different and that aligning program goals is a prerequisite for the successful implementation of patient-centered digital health services [63]. No studies included in this review made explicit use of co-design principles in intervention development using an established framework or theory, although a small number did incorporate end-user feedback at various stages. Few of the feasibility studies identified moved on to larger trials, supporting that uptake and integration of digital health interventions into usual clinical workflows remains problematic. Mounting evidence suggests that patient-orientated research—the inclusion of end-users in co-design and coproduction of interventions—assists in the generation of ideas and products that are feasible, appropriate, and of value to end-users [64,65]. Interventions designed to meet the requirements of end-users are associated with improved intervention acceptance, reduced user errors, and an enhanced reputation [65]. Evidence from other populations validates these points. For example, a co-design study of a flexible hip protector garment for older adults in care facilities resulted in high levels of interest from residents and support from site managers [66]. In another study, a codeveloped tool designed to improve the communication about heart failure trajectory and palliative care resulted in nurses reporting increased knowledge, improved confidence, and enhanced skills in end-of-life conversations [67]. Future work in digital health for children with special health care needs should incorporate co-design principles into the development of digital health interventions in order to increase user acceptance and intervention integration.

### Limitations of this Review

Although we attempted to be comprehensive in our search, missed studies may have limited the scope of this review. To be as comprehensive as possible, we followed a rigorous process using a predefined scoping methodology framework and assistance from an experienced librarian to develop our search strategy. We hand-searched reference lists of included articles and relevant journal databases to enhance the breadth of our search. However, we suspect that some organizations using digital health to care for children with special health care needs may be doing so without publishing their results. We did not contact experts in the field to inquire about known ongoing projects in this capacity; therefore, there is the possibility of some projects were missed.

Our team used an ongoing communication strategy, validation screening, and predefined study inclusion criteria and data extraction forms, contributing to the rigor of our data collection and extraction processes. However, due to time and resource constraints, we did not double screen the included studies. Thus, the potential for inappropriately including or excluding studies exists.

Additionally, we classified studies by methodology to the best of our ability, taking cues from authors’ own descriptions or stated study type. However, some studies had methodologies that were ambiguous or not well detailed, leading to difficulty in classifying them. We suggest that authors publishing future work on digital health intervention implementation use clear language and reference a well-developed model for intervention stage such as the NIH Stage Model for Behavioral Intervention Development [68].

Finally, as per scoping review methodology, no quality appraisal was conducted on the included studies. The intent of our review was a broad overview of the literature; thus, omitting a quality appraisal was appropriate, as we did not wish to exclude smaller or less rigorously conducted studies. However, because of this, we would caution readers who are intending to use the evidence from this review to conduct their own quality appraisal of individual studies. Although we have preidentified articles for a variety of children with special health care needs, the utilization of high-quality evidence in practice is of equal importance.

### Conclusions

The use of digital health to care for children with special health care needs presents an opportunity to leverage the capacity of technology to connect patients and their families to much-needed care from expert health care providers while avoiding the expenses and potential harms of the hospital-based care system. This review has summarized the use of digital health in providing care at home to children with special health care needs and their families while also highlighting challenges within the field. To move work in this important area forward, we strongly recommend the use of co-design and coproduction principles to involve end-users in meaningful ways in the design and implementation of digital health interventions. Additionally, much of the work in this area starts and ends with pilot and feasibility studies. Researchers should consider and integrate lessons learned from feasibility studies into large-scale interventions to operationalize programs with proven feasibility to better serve children with special health care needs and their families.

## Abbreviations

APN: advanced practice nurse
CF: cystic fibrosis
CRI: . chronic respiratory insufficiency
ED: emergency department
FO: family outcomes
HPO: health care provider outcomes
PO: patient outcomes
RN: registered nurse
SO: system outcomes
SW: social worker
